# Knee extensor strength normalised to body weight is associated with patient‐reported outcomes at 12 months after open‐wedge high tibial osteotomy

**DOI:** 10.1002/jeo2.70625

**Published:** 2026-02-02

**Authors:** Yuya Ueda, Takehiko Matsushita, Yohei Shibata, Ryo Goto, Daisuke Miura, Kumiko Ono, Akihiro Kida, Kyohei Nishida, Kanto Nagai, Yuichi Hoshino, Tomoyuki Matsumoto, Yoshitada Sakai, Ryosuke Kuroda

**Affiliations:** ^1^ Kobe University Graduate School of Health Sciences Kobe Japan; ^2^ Division of Rehabilitation Medicine Kobe University Hospital Kobe Japan; ^3^ Department of Orthopaedic Surgery Kobe University Graduate School of Medicine Kobe Japan; ^4^ Division of Rehabilitation Medicine Kobe University Graduate School of Medicine Kobe Japan

**Keywords:** knee extensor strength, knee osteotomy, open‐wedge high tibial osteotomy, patient‐reported outcomes, rehabilitation

## Abstract

**Purpose:**

Knee extensor strength is a critical factor in patients with knee osteoarthritis. However, no studies have investigated whether knee extensor strength directly affects patient‐reported outcomes after open‐wedge high tibial osteotomy (OWHTO). The purpose of this study was to investigate the association between knee extensor strength and patient‐reported outcomes after OWHTO.

**Methods:**

Patients who underwent OWHTO between 2016 and 2023 with knee function test results 12 months after surgery were included in this study. Isokinetic knee extensor strength was measured on the involved and uninvolved limb and normalised to body weight (KES/BW). Patient‐reported outcomes were assessed using the International Knee Documentation Committee (IKDC) subjective scores. Pearson correlation analysis and multivariable linear regression analysis were used to determine whether KES/BW on the involved limb was related to the IKDC subjective score.

**Results:**

Fifty‐eight knees from 52 patients who received OWHTO were evaluated. Pearson correlation analysis showed that KES/BW on the involved limb was significantly associated with the IKDC subjective score in OWHTO (*r* = 0.52, *p* < 0.001). Multivariable linear regression analysis indicated that KES/BW on the involved limb was independently associated with the IKDC subjective score at 12 months after OWHTO (*β* = 0.40, *p* = 0.02).

**Conclusions:**

KES/BW on the involved limb was independently associated with the IKDC subjective score at 12 months after OWHTO. This metric should be considered to achieve better patient‐reported outcomes after OWHTO.

**Level of Evidence:**

Level IV.

## Graphical abstract

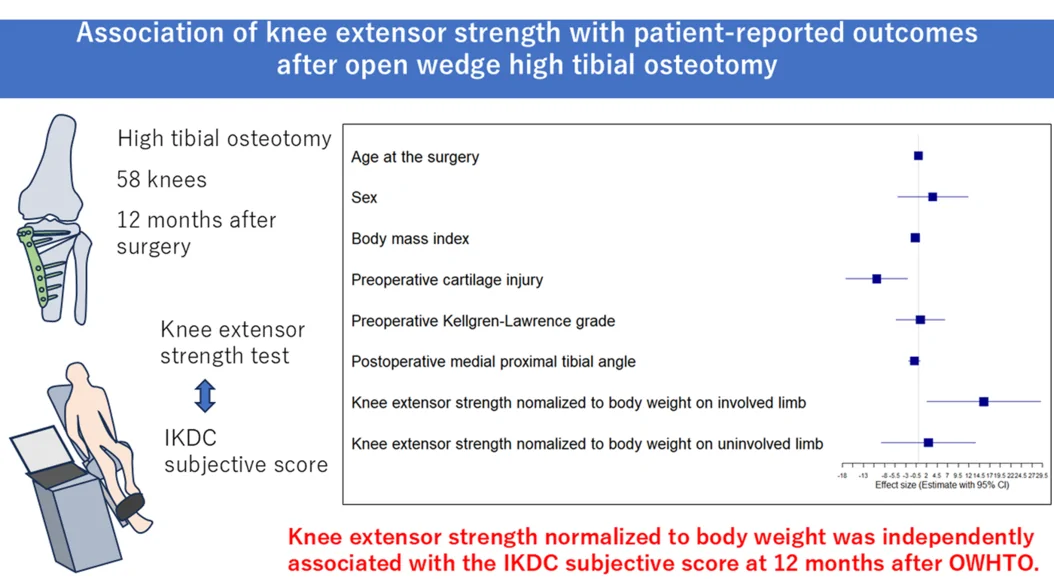

**Abbreviations**
AKO: around knee osteotomy
BMI: body mass index
BW: body weight
ICRS: International Cartilage Repair Society
IKDC: International Knee Documentation Committee; KES, knee extensor strength
KL: Kellgren–Lawrence
MA: mechanical axis
MPTA: medial proximal tibial angle
OA: osteoarthritis
OWHTO: open‐wedge high tibial osteotomy

## INTRODUCTION

Open‐wedge high tibial osteotomy (OWHTO) is widely performed to treat patients with medial compartmental knee osteoarthritis (OA) and previous studies have reported favourable outcomes including return to sports [6, 19, 24] and patient‐reported satisfaction [11, 25, 30]. Meanwhile, it has been reported that patient‐reported outcomes are affected by age [21], sex [7], body mass index (BMI) [5, 13], pre‐operative Kellgren–Lawrence (KL) grades [29] and post‐operative alignment of the knee [17, 20, 22]. Although these reports have indicated that patient background and surgical strategy could affect patient‐reported outcomes, the influence of post‐operative rehabilitation on the outcomes is poorly investigated.

Knee extensor strength is one of critical factors affecting physical function in patients with knee OA [4, 9] and it has been reported that knee extensor strength was associated with patient satisfaction 1 year after total knee arthroplasty [1, 8]. Notably, one study reported that post‐operative knee extensor strength on the involved limb was associated with patient‐reported outcomes after OWHTO [5], suggesting that knee extensor strength plats an important role in post‐operative outcomes after OWHTO. However, the study did not consider the effects of confounding factors, such as age, sex, OA grade of the knee and radiographic parameters. Therefore, whether knee extensor strength independently affects patient‐reported outcomes after OWHTO remains unclear and more comprehensive analysis is necessary to reveal the influence of knee extensor strength.

The aim of this study was to investigate the association between patient‐reported outcomes and knee extensor strength after OWHTO using multivariable analysis. It was hypothesised that knee extensor strength would be independently associated with patient‐reported outcomes after adjusting for confounding factors such as patient characteristics and post‐operative radiological parameters.

## METHODS

### Patient recruitment

This retrospective study was approved by the ethical review board of authors' affiliated institution. This study was performed according to the Declaration of Helsinki. Informed consent was obtained from all patients. Patients who underwent OWHTO between 2016 and 2023 and were assessed for knee function at our institution were included in this study. Patients who underwent concomitant ligament reconstruction and those with missing data were excluded. Surgical indications for OWHTO were varus knee alignment associated with medial compartmental osteoarthritis and/or cartilage injuries, and medial meniscus tears.

### Surgical procedures and rehabilitation

All surgeries were performed by four surgeons. OWHTO was performed as described in previous studies [2, 23]. The pes anserinus, including hamstring tendons, was detached from the insertion site and the superficial medial collateral ligament was released to expose the osteotomy site. Biplanar ascending osteotomy was performed in OWHTO. Two wedge‐shaped, β‐tricalcium phosphate blocks (Osferion60; OSferion Biomaterials Corp., Tokyo, Japan) were placed into the gap, depending on size. The detached hamstring tendons were reattached to the medial side of the tibial tuberosity to cover the osteotomy gap. A medial locking compression plate (TriS plate; OSferion Terumo Biomaterials Corp., Tokyo, Japan) was used to fix the tibia.

Post‐operative rehabilitation to improve knee range of motion and muscle strength began after 3 days, depending on whether the patient could tolerate the pain. After surgery, one‐third partial weight‐bearing was started 3 days after surgery and full weight‐bearing was permitted 3 weeks after surgery. If the cartilage treatment as mosaicplasty and autologous chondrocyte implantation or meniscal repair were added, the schedule for weight‐bearing was delayed by 2–4 weeks. Quadriceps strength training was started with caution because of the load on the osteotomy. Straight leg raise and leg extension exercise start immediately after surgery. Squatting started from 3 and 4 weeks postoperatively. The rehabilitation protocol was continued in an outpatient rehabilitation centre at a hospital or clinic after patient discharge.

### Measurements

Patient characteristics, including age at the time of surgery, sex, BMI, pre‐operative Tegner activity scale and concomitant medial or lateral meniscus injury requiring treatment (meniscectomy, centralisation or repair) and cartilage injury requiring treatment (abrasion, drilling, microfracture, mosaicplasty or autologous chondrocyte implantation) were recorded. Radiographic data, including pre‐operative KL grades, pre‐ and post‐operative mechanical axis (as percentages, % MA), and medial proximal tibial angle (MPTA), and joint line convergence angle (JLCA) were reviewed on anteroposterior long‐leg double‐standing radiographs.

**Figure 1:**
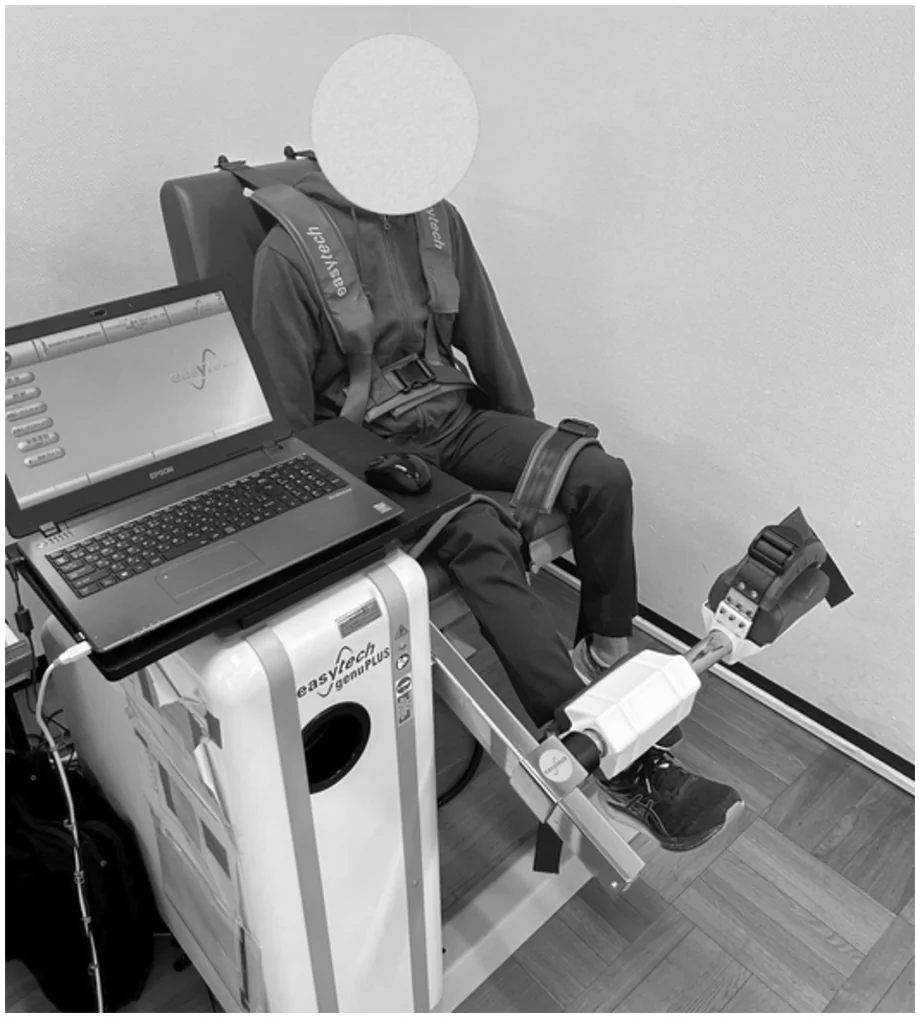
Isokinetic dynamometer.

Twelve months after surgery, knee function tests, including tests for knee extensor strength, were performed and patient‐reported outcomes were collected. Isokinetic quadriceps strength at 60°/s was assessed using an isokinetic dynamometer (Genu PLUS; Inter Reha Co., Ltd, Tokyo, Japan: Figure 1). The strength test was first performed with the uninvolved limb and then repeated on the involved limb. For patients who have previously undergone an around knee osteotomy (AKO) procedure on the contralateral side to the surgical side, the lower extremity that was operated on first was considered as the uninvolved side. Each participant performed two practice contractions, followed by five contractions at maximal effort, and the peak extension torque was measured. Knee extensor strength was expressed as percentage of body weight (KES/BW) and as the limb symmetry index (LSI). The KES/BW on each involved and uninvolved limb was calculated by normalising peak extension torque with BW (Nm/kg). LSI was calculated by normalising the peak torque of the involved limb versus that of the uninvolved limb and multiplying by 100.

The International Knee Documentation Committee (IKDC) subjective form was used to record patient‐reported outcomes. This joint‐specific outcome measure consists of 18 items; scores range from 0 to 100, with higher scores indicating better subjective knee function [14]. Patient scores were recorded preoperatively and postoperatively according to the previous reports [20, 22].

### Statistical analyses

All the analyses were performed using the data of each knee. If patient underwent HTO in both knees, the data of each knee was included separately.

The normality of each data distribution was determined by the Shapiro–Wilk test. Patient characteristics, radiographic data and the results of the knee function tests were described in knees after OWHTO. Pre‐ and post‐operative changes in the IKDC subjective scores and KES/BW on each involved and uninvolved limb were examined using the paired t‐test when pre‐operative data were available. Forty‐six knees that were available for both pre‐ and post‐operative IKDC subjective score were divided into two groups according to whether or not the knee achieved minimal clinically important difference (12.5 point) based on the previous report by Patel et al. [26] achievement or non‐achievement groups. The knee extensor strength data were compared between the two groups.

Pearson correlation analysis was performed to determine whether KES/BW on the involved and uninvolved limb and LSI values for knee extension strength were related to the IKDC subjective score.

Multivariable linear regression analysis was analysed to investigate whether KES/BW on the involved limb was associated with the IKDC subjective score. As covariates, age at the time of surgery, sex, BMI, pre‐operative KL grades, post‐operative MPTA, which have been reported in previous studies to be associated with patient‐reported outcomes after OWHTO, were included. Additionally, measurements that were significantly associated with IKDC subjective score in the univariable linear regression analysis were also entered as covariates. Finaly, a mixed‐effect model setting subject as a random effect was performed to analyse sensitivity since data of each knee from patients who received OWHTO in both knees were used separately.

The minimal sample size was calculated using G*Power (Version 3.1.9.4) to achieve an alpha level of 0.05 and a beta of 80% with a large effect size of 0.35 and considering the number of independent variables in the multivariable linear regression analysis. The minimum required sample size was 52 knees. All analyses were conducted using R version 4.1.1 (The R Foundation for Statistical Computing, Vienna, Austria).

## RESULTS

**Figure 2:**
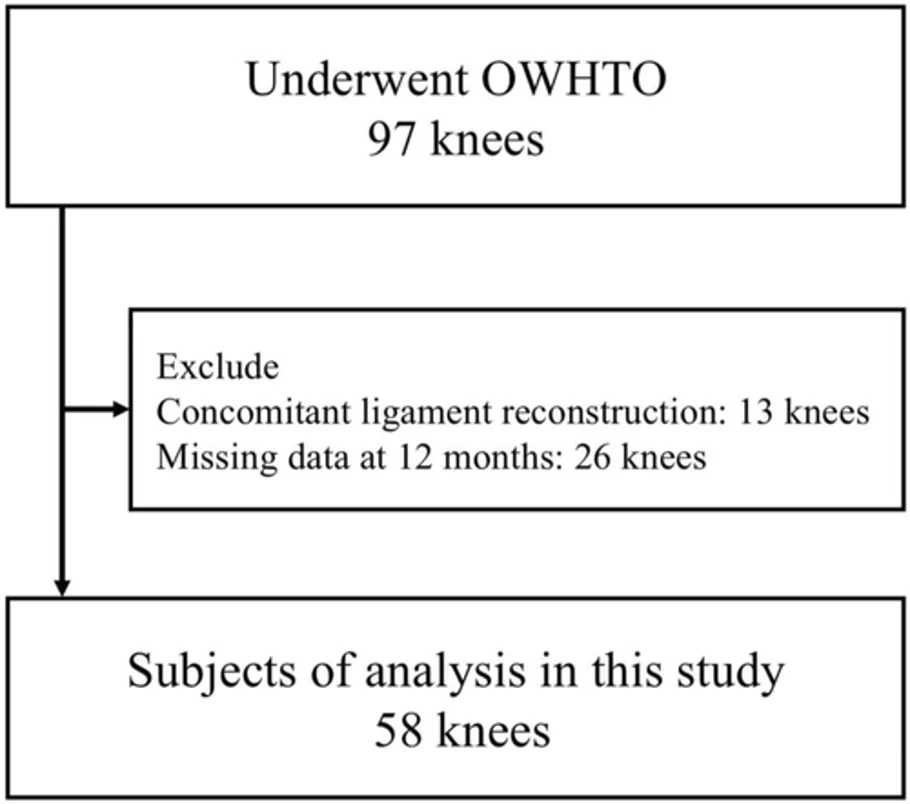
Flowchart of patient inclusion. OWHTO, Open‐wedge high tibial osteotomy.

This study included data from 58 knees surgeries from 52 patients (Table 1) (Figure 2). The data of 48 knees were for patients who underwent primary HTO surgery. The data of 10 knees were for patients who had already received AKO on the contralateral knee. In 10 contralateral HTO knees, the median days from initial AKO to contralateral HTO surgery was 672 days (Min–Max: 266–2205 days).

**Table 1:** Patient demographics, radiographic data and knee function test data.

	*N* = 58
Age at surgery, years	56.8 ± 10.1
Sex, female	32 (55.2%)
Body mass index, kg/m^2^	26.0 ± 3.6
Pre‐operative Tegner activity scale	3.9 ± 1.2
Medial meniscus injury, yes	47 (81.0%)
Lateral meniscus injury, yes	2 (3.4%)
Cartilage injury, yes	26 (44.8%)
Pre‐operative	
KL grades, 1/2/3/4	2/31/22/3
% MA, %	27.9 ± 11.2
MPTA, degree	85.4 ± 2.5
JLCA, degree	2.6 ± 1.3
KES/BW on involved limb,^a^ Nm/kg	0.9 ± 0.4
KES/BW on uninvolved limb,^a^ Nm/kg	1.3 ± 0.5
LSI for knee extensor strength,^a^ %	73.3 ± 28.6
IKDC subjective score^b^	41.4 ± 16.8
Post‐operative, 12 months	
% MA, %	57.6 ± 10.8
MPTA, degree	91.0 ± 2.7
JLCA, degree	1.7 ± 1.3
KES/BW on involved limb, Nm/kg	1.0 ± 0.4
KES/BW on uninvolved limb, Nm/kg	1.3 ± 0.5
LSI for knee extensor strength, %	84.2 ± 26.5
IKDC subjective score	62.0 ± 15.2
Primary OWHTO	48 (82.8%)

*Note*: Values are shown as the mean ± standard deviation or *N* (%) unless otherwise indicated. *p < 0.05.

Abbreviations: IKDC, International Knee Documentation Committee; JLCA, Joint line convergence angle; KES/BW, knee extensor strength as percentage of body weight; KL, Kellgren–Lawrence; % MA, percentage of mechanical axis; MPTA, medial proximal tibial angle; LSI, limb symmetry index; OWHTO, Open‐wedge high tibial osteotomy.

^a^ Data from 41 knees were available.

^b^ Data from 46 knees were available.

### Changes in knee extensor strength and patient‐reported outcomes 12 months after surgery

In 58 knees, pre‐operative data were available for 46 knees regarding IKDC (primary HTO, 36 knees; contralateral HTO, 10 knees) and for 41 knees regarding extensor strength (primary HTO, 32 knees; contralateral HTO, 9 knees). The paired t‐tests showed that KES/BW on the involved limb, LSI for knee extensor strength, and the IKDC subjective score significantly improved from the pre‐operative period to 12 months after surgery (Table 2).

**Table 2:** The results of paired t‐test between pre‐ and post‐operative change.

	Pre‐operative	Post‐operative	*p* value
KES/BW on involved limb,^a^ Nm/kg	0.9 ± 0.4	1.1 ± 0.4	0.003*
KES/BW on uninvolved limb,^a^ Nm/kg	1.3 ± 0.5	1.3 ± 0.5	0.17
LSI for knee extensor strength,^a^ %	73.3 ± 28.6	86.4 ± 26.7	0.02*
IKDC subjective score^b^	41.4 ± 16.8	61.6 ± 15.6	<0.001*

Abbreviations: IKDC, International Knee Documentation Committee; KES/BW, knee extensor strength as percentage of body weight; LSI, limb symmetry index.

* *p* < 0.05.

^a^ Data from 41 knees were available.

^b^ Data from 46 knees were available.

The achievement and non‐achievement groups included 28 knees and 18 knees respectively. The mean knee extensor strength on involved and uninvolved limbs in the achievement group were significantly higher than those in the non‐achievement group (1.1 ± 0.5 Nm/kg vs. 0.9 ± 0.2 Nm/kg, *p* = 0.012; 1.4 ± 0.5 Nm/kg vs. 1.1 ± 0.3 Nm/kg, *p* = 0.012), respectively) (Table 3).

**Table 3:** The comparison of knee extensor strength between the MCID achievement and the non‐achievement group.^a^

	The non‐achievement group, *N* = 18	The achievement group, *N* = 28	*p* value
Pre‐operative			
KES/BW on involved limb, Nm/kg	0.8 ± 0.4^b^	0.9 ± 0.4^c^	0.49
KES/BW on uninvolved limb, Nm/kg	1.2 ± 0.5^b^	1.3 ± 0.5^c^	0.33
LSI for knee extensor strength, %	74.3 ± 29.6^b^	74.5 ± 27.6^c^	0.98
Post‐operative			
KES/BW on involved limb, Nm/kg	0.9 ± 0.2	1.1 ± 0.5	0.012*
KES/BW on uninvolved limb, Nm/kg	1.1 ± 0.3	1.4 ± 0.5	0.02*
LSI for knee extensor strength, %	86.3 ± 35.1	82.2 ± 23.4	0.67

Abbreviations: KES/BW, knee extensor strength as percentage of body weight; LSI, limb symmetry index; MCID, minimal clinically important difference.

* *p* < 0.05.

^a^ Data from 46 knees were available.

^b^ Data from 17 knees were available.

^c^ Data from 23 knees were available.

### Association of knee extensor strength with IKDC subjective scores

**Figure 3:**
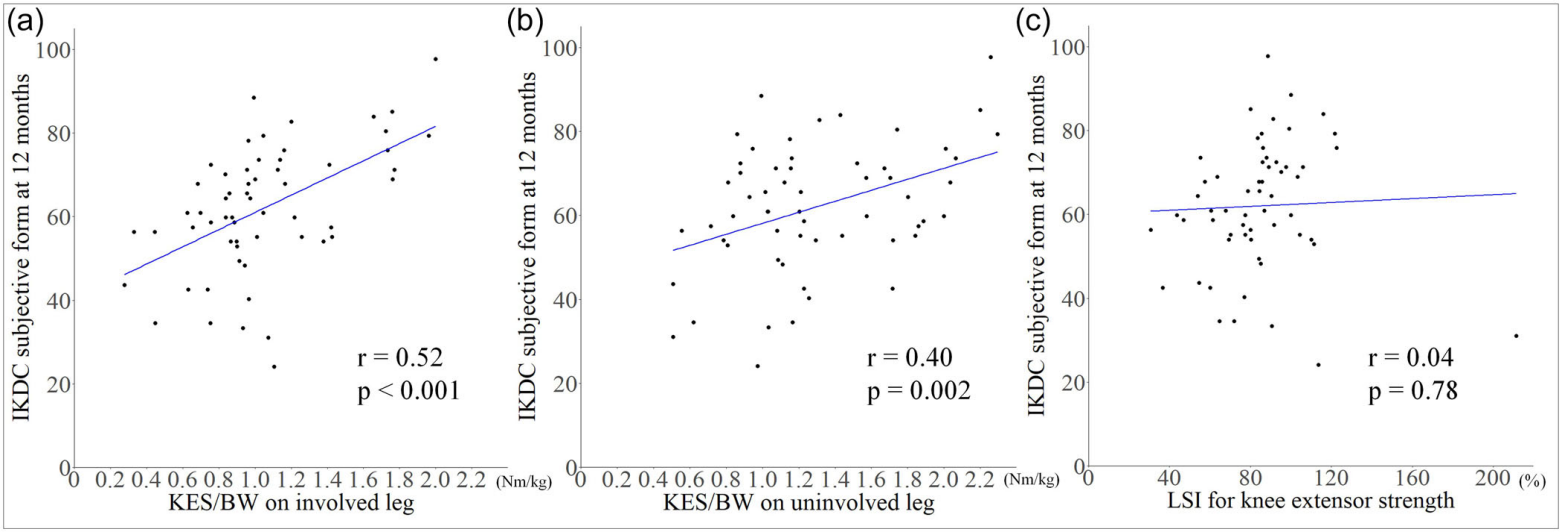
Pearson correlation analysis between the IKDC subjective score and (a) KES/BW on the involved limb, (b) KES/BW on the uninvolved limb, and (c) LSI for knee extensor strength. IKDC, International Knee Documentation Committee; KES/BW, knee extensor strength as percentage of body weight; LSI, limb symmetry index.

Pearson correlation analysis showed that KES/BW on each involved (*r* = 0.52, *p* < 0.001) and uninvolved limb (*r* = 0.40, *p* = 0.002) were significantly associated with the IKDC subjective score at 12 months. In contrast, the LSI for knee extensor strength was not associated with the IKDC subjective score (*r* = 0.04, *p* = 0.76) (Figure 3).

Multivariable linear regression analysis (adjusted *R* ^2^ = 0.36) indicated that KES/BW on the involved limb (*β* = 0.40, *p* = 0.02) and cartilage injury (*β* = −0.33, *p* = 0.009) were independently associated with the IKDC subjective score 12 months after OWHTO (Table 4).

**Table 4:** The univariate and multivariable linear regression analysis for the IKDC subjective score.

	Univariable analysis			Multivariable analysis		
	*β*	95% CI	*p* value	*β*	95% CI	*p* value
Age at surgery	−0.07	−0.33, 0.20	0.63	0.03	−0.23, 0.28	0.83
Sex, female	−0.18	−0.44, 0.09	0.19	0.11	−0.16, 0.38	0.42
Body mass index	−0.34	−0.59, −0.08	0.01*	−0.17	−0.40, 0.07	0.16
Pre‐operative Tegner activity scale	0.05	−0.22, 0.32	0.71	–	–	–
Medial meniscus injury, yes	0.19	−0.07, 0.45	0.15	–	–	–
Lateral meniscus treatment	−0.14	−0.41, 0.12	0.29	–	–	–
Cartilage injury, yes	−0.48	−0.71, −0.24	<0.001*	−0.33	−0.57, −0.09	0.009*
Pre‐operative KL grades	−0.13	−0.40, 0.13	0.32	0.02	−0.22, 0.27	0.85
Post‐operative % MA	0.03	−0.24, 0.30	0.84	–	–	–
Post‐operative MPTA	−0.14	−0.40, 0.13	0.31	−0.16	−0.40, 0.08	0.18
Post‐operative JLCA	−0.21	−0.47, 0.05	0.12	–	–	–
KES/BW on involved limb	0.52	0.30, 0.75	<0.001*	0.40	0.06, 0.74	0.02*
KES/BW on uninvolved limb	0.40	0.15, 0.65	0.002*	0.07	−0.27, 0.41	0.68
LSI for knee extensor strength	0.04	−0.23, 0.31	0.76	–	–	–
Primary OWHTO, yes	0.01	−0.26, 0.27	0.97	–	–	–

*Note*: Adjusted *R* ^2^ for multivariable analysis was 0.36.

Abbreviations: CI, confidence interval; IKDC, International Knee Documentation Committee; JLCA, Joint line convergence angle; KES/BW, knee extensor strength as percentage of body weight; KL, Kellgren–Lawrence; LSI, Limb symmetry index; % MA, percentage of mechanical axis; MPTA, medial proximal tibial angle; OWHTO, Open‐wedge high tibial osteotomy.

* *p* < 0.05.

**Figure 4:**
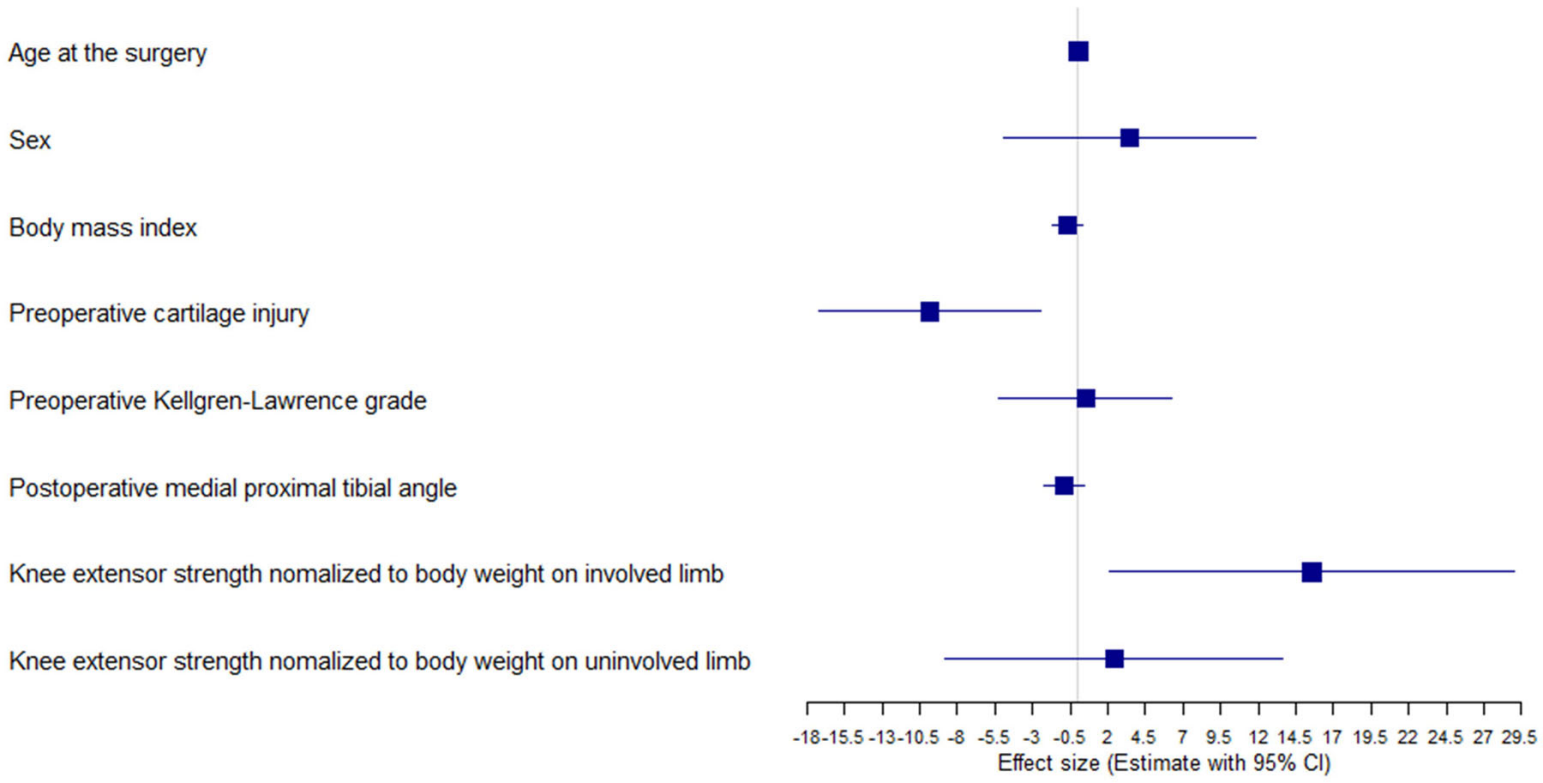
Forest plot of the mix‐effect model results.

Consitentl with the multivariable analysis, the sensitivity analysis using a mixed‐effect model (Conditional *R* ^2^; 0.43, Marginal *R* ^2^; 0.41) showed that KES/BW on the involved limb (Estimate = 15.6, *p* = 0.02) and cartilage injury (estimate = −9.8, *p* = 0.009) were independently associated with the IKDC subjective score 12 months after OWHTO (Figure 4). The detailed results are shown in Supporting Information: Appendix 1.

## DISCUSSION

The finding of the present study is that knee extensor strength on the involved limb was independently associated with the IKDC subjective score 12 months after OWHTO. This result suggests that knee extensor strength is an important measure to improve patient‐reported outcomes 12 months after OWHTO. Thus, rehabilitation to improve this variable factor may be effective in improving patient‐reported outcomes after OWHTO.

A previous study revealed that knee extensor strength was a critical variable associated with improved patient‐reported outcomes after knee arthroplasty [8]. However, few studies have investigated these associations in relation to knee osteotomy. Azuma et al. reported that knee extension strength correlated with the Japanese Orthopedic Association score regarding the knee joint at 12 months after OWHTO [5]. Similar results were obtained in the present study, in that KES/BW on the involved limb correlated with the IKDC subjective score after each OWHTO. In addition, these associations were not affected after adjusting for covariates such as age, BMI and some radiographic data. The results of our study suggest that post‐operative knee extensor strength on the involved limb is independently related to patient‐reported outcomes after OWHTO. Therefore, specific rehabilitation programs may be needed to optimise recovery of quadriceps muscle strength after OWHTO.

The LSI is used as an indicator of knee function recovery after orthopedic surgery for lower extremity sports injuries such as anterior cruciate ligament reconstruction [10]. The limited data in the present study showed that the LSI for knee extensor strength improved from pre‐operative to post‐operative at 12 months after OWHTO. Goto et al. also reported that knee extensor strength was significantly recovered after OWHTO [12]. However, the LSI for knee extensor strength was not associated with the IKDC subjective score in the patients who underwent OWHTO. Knee muscle weakness in the lower limbs bilaterally in patients with knee osteoarthritis [3, 15]. These results indicate that LSI may not be an adequate measure of the recovery of knee extensor strength after OWHTO in patients with functional loss in the uninvolved knee joint. However, as this study included patients who had undergone bilateral knee osteotomy but did not evaluate contralateral knee osteoarthritis, future studies are needed to determine the usefulness of LSI after osteotomy around the knee.

This study also revealed that pre‐operative cartilage injury requiring treatment was negatively associated with the IKDC subjective score. Spahn et al. reported that degree IV cartilage defect of the tibia was a significant pre‐operative factor for predicting a poor outcome after HTO [28]. Jin et al also reported that intraoperative medial compartment cartilage injury the International Cartilage Repair Society (ICRS) ≥ Grade 4 and lateral compartment cartilage injury ICRS ≥ 2 were associated with failure (the conversion to total knee arthroplasty or Knee Society Score of < 60 points) after OWHTO [16]. In addition, Schröter et al. showed that early full weight‐bearing after OWHTO leads to earlier improvement of the Lysholm score [27]. This study may explain the negative impact of cartilage injury on IKDC subjective score in this study. Controversially, cartilage regeneration in medial compartment after OWHTO has been described [18], and that can affect improvement clinical outcomes after OWHTO. This study did not investigate cartilage regeneration by second look. Therefore, the influence of cartilage injury on patient‐reported outcomes needs to be clarified in future studies.

This study had some limitations. First, as this was a cross‐sectional single‐centre study, causality and generalisability should be considered with caution. In addition, the analysis did not consider changes in limb alignment and knee extensor strength from pre‐ to post‐operative, which warrant examination in longitudinal studies. Second, due to the sample size, inclusion of patients who underwent bilateral surgery raised some issues. Further studies are needed to investigate only patients with primary OWHTO. Third, at approximately 12 months, the post‐operative follow‐up duration was short. Medium‐ to long‐term evaluations are also required to demonstrate the utility of knee extensor strength.

## CONCLUSION

KES/BW on the involved limb was independently associated with the IKDC subjective score in patients who underwent OWHTO. Knee extensor strength on the involved limb should be considered to achieve better patient‐reported outcomes after OWHTO.

## AUTHOR CONTRIBUTIONS

Yuya Ueda was involved in the conception and design of the study, the acquisition, analysis and interpretation of the data, and writing the article. Takehiko Matsushita was involved in the conception and design of the study, development of the research, and writing the article. Yohei Shibata, Ryo Goto, Daisuke Miura, Kumiko Ono, Akihiro Kida, Kyohei Nishida, Kanto Nagai, Yuichi Hoshino, Tomoyuki Matsumoto, Yoshitada Sakai, and Ryosuke Kuroda were involved in the acquisition and interpretation of the data. All of the authors were involved in the critical revisions of the article for its important intellectual content, and they all approved the final version of the article.

## CONFLICT OF INTEREST STATEMENT

The authors declare no conflicts of interest.

## ETHICS STATEMENT

Institutional review board approval was obtained from the Kobe University ethics committee (approval number: 170176). This study was performed according to the Declaration of Helsinki. All patients provided informed consent.

## Supporting information

Appedix file 1.

## Data Availability

Data supporting the findings of this study are available from the corresponding author on reasonable request.
