# Supplementary material for: Knee extensor strength normalised to body weight is associated with patient‐reported outcomes at 12 months after open‐wedge high tibial osteotomy
Source: J Exp Orthop. 2026 Feb 2;13(1):e70625. doi: 10.1002/jeo2.70625 (PMC12863416; doi:10.1002/jeo2.70625)
Supplement: Supplementary file 1 — Appedix file 1. [file JEO2-13-e70625-s001.docx]

Appendix file 1

| Appendix 1. The result of mixed-effects model as a sensitivity analysis. | | | |
| --- | --- | --- | --- |
|  | Estimate | t value | p value |
| Age at surgery | 0.04 | 0.23 | 0.82 |
| Sex, female | 3.45 | 0.84 | 0.41 |
| Body mass index | -0.70 | -1.43 | 0.16 |
| Cartilage injury, yes | **-9.82** | **-2.69** | **0.01*** |
| Preoperative KL grades | 0.52 | 0.18 | 0.86 |
| Postoperative MPTA | -0.91 | -1.34 | 0.19 |
| KES/BW on involved limb | **15.56** | **2.33** | **0.02*** |
| KES/BW on uninvolved limb | 2.43 | 0.44 | 0.66 |
| * p < 0.05; The conditional R^2^ was 0.43 and the marginal R^2^ was 0.41; | | | |
| KL, Kellgren–Lawrence; MPTA, medial proximal tibial angle; | | | |
| KES/BW, knee extensor strength as percentage of body weight; | | | |
| IKDC, International Knee Documentation Committee. | | | |
